# Rac‐GTPases and Rac‐GEFs in neutrophil adhesion, migration and recruitment

**DOI:** 10.1111/eci.12939

**Published:** 2018-05-11

**Authors:** Chiara Pantarelli, Heidi C. E. Welch

**Affiliations:** ^1^ Signalling Programme Babraham Institute Cambridge UK

**Keywords:** guanine‐nucleotide exchange factors, neutrophils, P‐Rex1, Rho‐GTPases, small G proteins, Vav

## Abstract

Rac‐GTPases and their Rac‐GEF activators play important roles in the recruitment and host defence functions of neutrophils. These proteins control the activation of adhesion molecules and the cytoskeletal dynamics that enable the adhesion, migration and tissue recruitment of neutrophils. They also regulate the effector functions that allow neutrophils to kill bacterial and fungal pathogens, and to clear debris. This review focuses on the roles of Rac‐GTPases and Rac‐GEFs in neutrophil adhesion, migration and recruitment.

## 1 NEUTROPHIL FUNCTIONS

During inflammation, neutrophils are rapidly recruited from the blood stream into inflamed and infected tissues. There they release proinflammatory mediators to attract other inflammatory cells and mount effector functions to kill pathogens.^1^, ^2^ For recruitment, neutrophils adhere to the inflamed blood vessel wall and migrate through it into inflamed tissues.^1^ As the fastest migrating vertebrate cell type,^3^ they are the rapid‐response unit of the immune system. Once arrived at the source of inflammation, they mount pathogen‐ and debris‐clearing effector functions, including degranulation, phagocytosis and the production of both reactive oxygen species (ROS) and neutrophil extracellular traps (NETs).^2^ Human leucocyte adhesion deficiency and neutrophil immunodeficiency syndrome, conditions characterized by severe recurrent infections and poor wound healing, are evidence for the importance of neutrophil recruitment and effector responses in host defence.^4^, ^5^, ^6^ Yet neutrophils must be tightly regulated; excessive recruitment and activity exacerbate inflammation and cause tissue injury (references 1, 2, 7; see also the article by Gomez et al in this issue).

## 2 NEUTROPHIL RECRUITMENT

**Figure 1:**
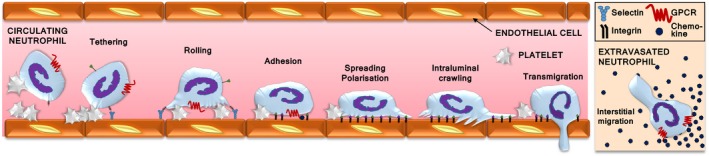
Neutrophil recruitment. Neutrophil recruitment from the blood stream into inflamed and infected tissues begins with the upregulation of selectins on the surface of vascular endothelial cells and circulating platelets. Selectin‐dependent interactions of neutrophils with platelets and vascular endothelial cells enable the tethering and rolling of neutrophils along the vascular endothelium. GPCR signalling activates integrins, which bring about neutrophil arrest on the vascular wall, followed by firm adhesion, spreading and polarization, intravascular crawling and transendothelial migration. Neutrophils use amoeboid chemotaxis to follow chemokine gradients within the interstitium towards the source of the inflammation

Neutrophil recruitment into inflamed and infected tissues proceeds in well‐defined steps^1^ (Figure 1). First, the upregulation of adhesion molecules on the surface of vascular endothelial cells and circulating platelets renders these cells sticky.^1^, ^8^ Selectins enable the loose tethering and rolling of neutrophils on the vascular endothelium, in effect snatching the neutrophils from the fast‐flowing circulation. Neutrophil G‐protein‐coupled receptors (GPCRs) are activated by inflammatory chemokines trapped on the vascular surface.^9^ This activation induces the upregulation and opening of integrins, which confer neutrophil arrest, firm adhesion and intravascular crawling on the vascular endothelium. Neutrophils then traverse the endothelial cell layer (via para‐ or transcellular routes), the basal lamina and the abluminal pericyte layer by integrin‐dependent migration. Depending on the tissue, they follow chemokine gradients, using either amoeboid (integrin‐independent) or integrin‐dependent chemotaxis to migrate through the interstitium.^10^ Finally, they reach the source of inflammation or infection.

## 3 RAC‐GTPASES AND THEIR GEFS IN NEUTROPHILS

**Figure 2:**
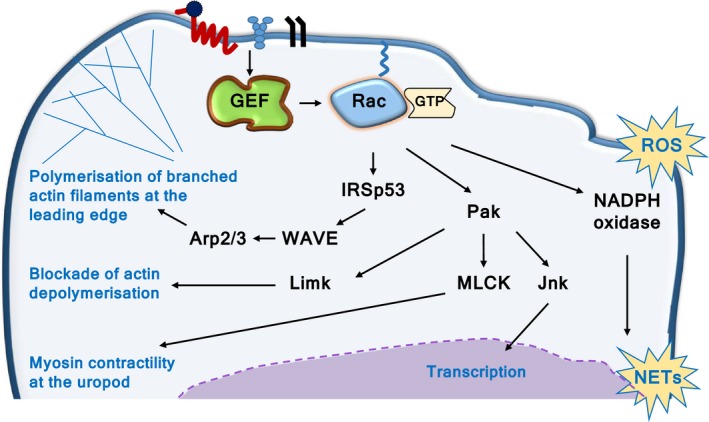
Rac in neutrophil adhesion and migration**.** Rac‐GTPases control actomyosin cytoskeletal dynamics in several ways, including the IRSp53, WAVE, Arp2/3 pathway, which induces the polymerization of branched actin filaments at the cell periphery to enable firm adhesion and spreading. Localized Rac activity induces leading edge formation and polarization. In addition, Rac blocks actin depolymerization through the Limk pathway and stimulates myosin contractility at the uropod through MLCK. Combined, these pathways stabilize polarity and enable migration, as well as other processes such as degranulation and phagocytosis. In addition, active Rac is an integral subunit of the NADPH oxidate complex, which produces ROS and thus controls the ROS‐dependent production of NETs. Finally, Rac also controls gene expression through Jnk

Rac proteins are small guanine‐nucleotide binding proteins (G proteins, GTPases) of the Rho family.^11^, ^12^ Neutrophils express the ubiquitous isoform Rac1, hematopoietic Rac2 and widely expressed RhoG, but not the neuronal isoform Rac3.^13^ These GTPases control the structure of the actomyosin cytoskeleton by signalling through several pathways, mainly through IRSp53, WAVE and Arp2/3, thus enabling the polymerization of branched actin filaments at the cell periphery. This and other Rac signalling pathways that control actomyosin cytoskeletal dynamics are central for neutrophil adhesion, spreading and the formation of a leading edge that confers cell polarization and migration^14^, ^15^ (Figure 2). Indeed, the use of photoactivatable Rac has shown that localized activation of Rac at the leading edge is sufficient for directional neutrophil migration in zebrafish.^16^ In addition, Rac‐GTPases control other responses that require cytoskeletal dynamics, such as integrin‐dependent phagocytosis and the degranulation of azurophil granules. Furthermore, active Rac2 is an integral part of the NADPH oxidase (NOX2) enzyme complex and is thus directly involved in ROS production. In turn, ROS is required to make NETs, and both are crucial responses for killing pathogens [Van Avondt & Hartl, this issue]. Finally, Rac also regulates gene expression through kinases such as Pak and Jnk, which signal to a range of downstream effectors, including transcription factors.^13^ The signalling pathways and functions of Rac‐GTPases in neutrophils are broadly recapitulated in neutrophil‐like cell lines (such as HL60 and NB4), but there are significant differences, such as in migratory behaviour. Hence, we focus here on primary neutrophils.

**Figure 3:**
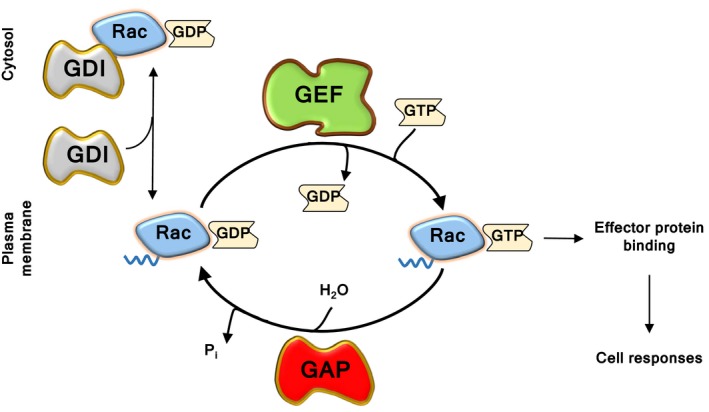
Regulation of Rac activity**.** Rac‐GTPases cycle between their GTP‐bound active and GDP‐bound inactive form. They are activated by guanine‐nucleotide exchange factors (GEFs), which remove GDP, thus enabling excess cellular GTP to bind. The binding of active Rac to downstream effector proteins elicits cell responses. GTPase‐activating proteins (GAPs), which increase the GTPase activity of Rac, are the off‐switch. Inactive Rac is sequestered in the cytosol by guanine‐nucleotide dissociation inhibitors (GDIs)

Like most small GTPases, Rac proteins are molecular switches, active when GTP‐bound and inactive when GDP‐bound^11^ (Figure 3). They are activated when guanine‐nucleotide exchange factors (GEFs) remove GDP, thus allowing excess free cellular GTP to bind to Rac.^17^, ^18^ In their active GTP‐bound conformation, Rac‐GTPases engage target proteins that transmit signals downstream. Deactivation of Rac occurs by GTP hydrolysis through its GTPase activity, which is enhanced by GTPase‐activating proteins (GAPs).^17^ Additional regulation comes from guanine‐nucleotide dissociation inhibitors (GDIs), which bind C‐terminal prenylated residues of Rac, thus sequestering the inactive GTPase in the cytosol. Our review describes the roles of Rac‐GTPases and their GEF activators in neutrophils. For related reviews that discuss neutrophil GAPs, the proteins that switch off GTPase signalling (please see references 13, 19; and Csépányi‐Kömi et al in this issue).

**Figure 4:**
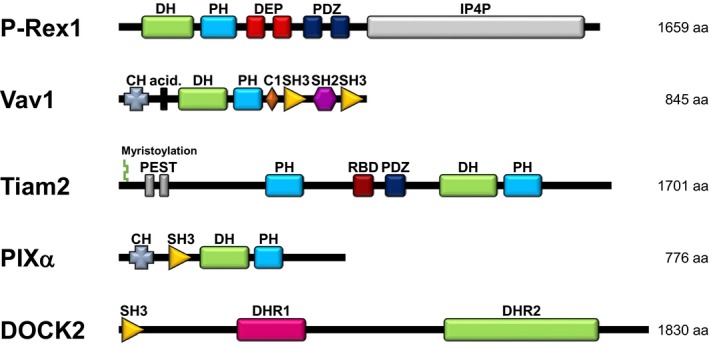
Neutrophil Rac‐GEFs. Neutrophils express several types of Dbl‐type and DOCK‐type Rac‐GEFs. The Dbl‐type Rac‐GEFs, which all feature the typical catalytic DH domain and tandem PH domain, include P‐Rex1, the 3 Vav family Rac‐GEFs (Vav1, Vav2, Vav3) and Tiam2. The Rac‐ and Cdc42‐GEF PIXα is also expressed, but to date, only its Cdc42‐GEF activity has been observed directly. The DOCK‐type Rac‐GEFs, which signal through a DHR2 catalytic domain, include DOCK2 and DOCK5. The precise structure of their catalytic domain determines which Rac isoform the GEFs can activate. The multidomain structure unique to each type of Rac‐GEF couples these proteins to distinct sets of regulators and effectors, ensuring the activation of GEFs within specific signalling networks and thus enabling the activation of selected subsets of Rac responses. The GEF domains that confer this specificity include protein‐binding domains, such as SH3 and PDZ, and lipid‐binding domains such as extra PH domains outside of the catalytic DH/PH tandem

Multiple types of Rac‐GEFs are usually expressed within each cell type. Neutrophil Rac‐GEFs include proteins from the Dbl‐type P‐Rex, Vav and Tiam families, and from the structurally unrelated DOCK family (Figure 4). These GEFs show substrate preferences for different Rac isoforms, determined by the precise structure of their catalytic DH or DHR2 domains (for Dbl and DOCK‐type Rac‐GEFs, respectively). Some Rac‐GEFs can also activate other Rho‐GTPases, for example Vav, which can activate RhoA as well as Rac. In addition to their catalytic domains, Rac‐GEFs have varied multidomain structures that couple each GEF to specific upstream and effector proteins. Together, these mechanisms determine which Rac‐dependent cell responses ensue.^13^, ^20^ Therefore, the activity of Rac‐GEFs must be acutely and tightly regulated, through a combination of mechanisms, including phosphorylation, protein and lipid binding, unique to each type of GEF.^18^, ^20^

A central mechanism of regulation is the lipid second messenger phosphatidylinositol‐(3,4,5)‐trisphosphate (PIP_3_), produced by phosphoinositide 3‐kinase (PI3K) within the cell membrane.^19^, ^20^ Without PIP_3_, neutrophils cannot generate stable polarity and migration.^21^, ^22^ PIP_3_ localizes several Rac‐GEFs and other signalling proteins to the plasma membrane and activates some directly, by binding to their PH domain. This enables activation of Rac at the cell periphery to confer firm adhesion and spreading. Polarized production of PIP_3_ and activation of Rac induce the formation of a leading edge, cell polarization and migration.^15^ Importantly, PIP_3_‐dependent regulation of neutrophil Rac‐GEFs always occurs in conjunction with other signalling mediators unique to each GEF.

**Figure 5:**
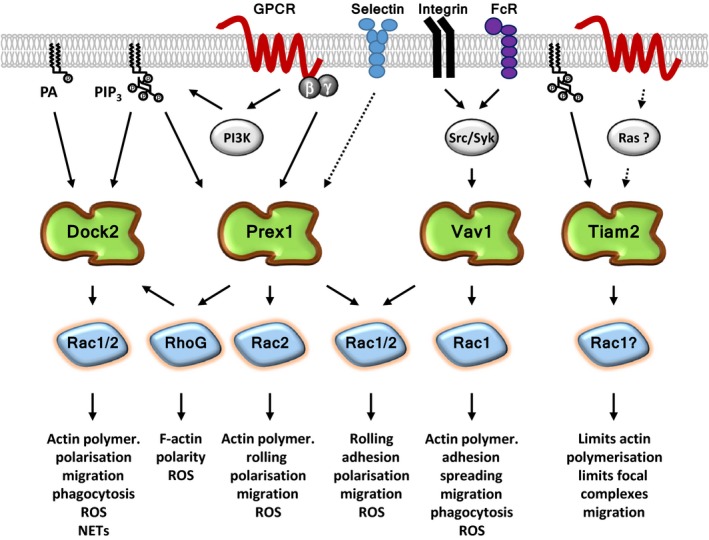
Signalling pathways of neutrophil Rac‐GEFs. The Rac‐GEF P‐Rex1, which mediates signalling through GPCRs, E‐selectin and TLR4 (not all shown here for simplicity), is activated by the lipid second messenger PIP_3_ and by the Gβγ subunits of heterotrimeric G proteins. The Vav family Rac‐GEFs, which are activated by tyrosine phosphorylation, are important in integrin and FcR signalling, but they also couple to TLR4 and GPCRs. It is currently unknown, which mechanisms control Tiam2 in neutrophils, except that this GEF controls chemoattractant‐induced responses. DOCK2 also signals upon GPCR stimulation. It is activated by RhoG and recruited to the plasma membrane by PIP_3_ and phosphatidic acid (PA). The preferred Rac substrate of each Rac‐GEF is shown here, but usually, the GEFs can activate both Rac1 and Rac2 to some extent. P‐Rex1 can also activate RhoG and may thus signal in sequence with DOCK2 in some pathways. The list of cell responses regulated by each neutrophil Rac‐GEF is likely to grow with further study

The Rac‐GEF P‐Rex1 is known to signal downstream of GPCRs, E‐selectin and toll‐like receptor 4 (TLR4) in mouse neutrophils.^23^ It is directly activated by PIP_3_ and also by the Gβγ subunits of heterotrimeric G proteins, which are released upon activation of GPCRs. In addition, various phosphorylation events modulate its activity^23^, ^24^, ^25^ (Figure 5). PIP_3_ and Gβγ subunits also recruit this GEF to the plasma membrane,^25^, ^26^, ^27^ possibly in conjunction with the GPCR‐adaptor protein Norbin, although this remains to be tested in neutrophils.^28^ Like P‐Rex1, the Rac‐GEF DOCK2 signals in response to GPCR stimulation. However, in contrast to P‐Rex1, DOCK2 is activated by the binding of active RhoG to its adaptor protein ELMO, and it is recruited to the plasma membrane by PIP_3_ and phosphatidic acid, a product of phospholipase D or diacyl‐glycerol kinase activity.^29^, ^30^ Differently again, Vav family Rac‐GEFs (of which Vav1 and Vav3 are most prominently expressed in neutrophils) are activated by protein tyrosine kinases downstream of various types of receptors, including integrins, Fc receptors (FcR), GPCRs and TLR4.^31^ Least is currently known about the Rac‐GEF Tiam2, which was described fairly recently in neutrophils.^32^ Tiam‐family Rac‐GEFs are generally directly activated by Ras and modulated by a variety of mechanisms, including phosphorylation, and they translocate to the plasma membrane upon binding PIP_3_.^33^ In neutrophils, Tiam2 was shown to regulate chemoattratant‐stimulated responses.^32^ However, the mechanisms of Tiam2 regulation in these cells remain to be elucidated.

## 4 RAC‐GTPASES IN NEUTROPHIL ADHESION, MIGRATION AND RECRUITMENT

Human neutrophil immunodeficiency syndrome is caused by a dominant‐negative Rac2 mutation (D57N). This mutation renders Rac2 unable to bind GTP but still able to bind Rac‐GEFs, thus forming an unproductive complex that cannot exchange GDP for GTP, and thereby sequestering upstream signals without transducing them further.^5^ This Rac2 mutation impairs the L‐selectin‐dependent rolling, integrin‐dependent adhesion and chemotaxis of neutrophils.^5^, ^6^, ^34^, ^35^ In zebrafish neutrophils, introduction of this Rac2 mutation as a transgene largely recapitulates the human disease; neutrophil motility and tissue recruitment are impaired, and fish larvae are less likely to survive infection with *P. aeruginosa*.^36^ In addition to this dominant‐negative mutation, a loss‐of‐function mutation of Rac2 (W56X) was recently identified in patients with a form of common variable immunodeficiency. Neutrophils from these patients show reduced chemotaxis and abnormalities in their secretory granules,^37^ but further in‐depth analysis remains to be performed. Rac2^−/−^ mouse neutrophils show reduced actin polymerization, L‐selectin‐dependent rolling and integrin‐dependent spreading and are unable to form stable leading edges and thus cannot migrate.^5^, ^38^, ^39^ Consequently, neutrophil recruitment is reduced in Rac2^−/−^ mice during sterile peritonitis^5^, ^38^ and during immune‐complex induced acute lung injury.^40^ Rac2^−/−^ zebrafish larvae also show reduced neutrophil recruitment to cut wounds and poor immunity against *P. aeruginosa* infections.^41^ Furthermore, there is a clear gene‐dosage effect of Rac2 expression in neutrophils; heterozygous Rac2^+/−^ mouse neutrophils chemotax less well than wild‐type but better than Rac2^−/−^ cells.^42^ Similarly, partial suppression of Rac2 levels by overexpression of microRNA‐722 in zebrafish larvae reduces neutrophil recruitment to sites of tissue injury. Unexpectedly, this also increases larval survival upon endotoxin challenge or *P. aeruginosa* infection, whereas knockout studies would have predicted worse outcomes. The authors argue that such partial suppression of Rac2 may retain immune functions while preventing excessive inflammation.^43^ Further corroboration of this hypothesis would be useful. In principle, it is imaginable that a drug could be developed which fine‐tunes Rac2 to levels that preserve immunity but reduce neutrophil‐dependent inflammatory conditions.

In contrast to Rac2, no Rac1 mutation is known to cause human immunodeficiency. Interestingly, reduced levels of Rac1 (and other Rho‐GTPases and their regulators) were recently linked to the upregulation of microRNAs in human myelodysplastic syndrome, a condition characterized by a range of functional neutrophil defects.^44^ However, more study is required to establish causal relationships and specificity. Deleting Rac1 in the mouse is embryonic lethal, but drug‐inducible conditional Rac1‐deficiency is known to increase the integrin‐mediated spreading of neutrophils derived from hematopoietic stem cells.^39^ Myeloid‐lineage specific Rac1 deletion reduces actin polymerization and the efficiency of chemotaxis,^45^ the latter by affecting directionality^46^ and uropod retraction.^47^ In vivo, conditional Rac1‐deficiency impairs neutrophil recruitment during sterile peritonitis^45^ and acute fMLP‐induced lung inflammation.^48^ Combined deficiency of Rac1 and Rac2 has more severe effects. It abolishes neutrophil recruitment to lungs infected with *E. coli*.^49^ It also delays recruitment into the synovial fluid of inflamed joints in an arthritis model triggered by *C. trachomatis* infection. This ameliorates the acute phase but causes more severe disease during the chronic phase.^50^

The Rac‐GTPase RhoG can signal upstream of Rac1 and Rac2, at least in some pathways (see below), and might therefore be expected to have similar importance for neutrophil adhesion and migration. Indeed, RhoG does contribute to full polarization of actin filaments at the leading edge of chemoattractant‐stimulated neutrophils.^30^ However, unlike Rac1 and Rac2, RhoG is dispensible for the chemoattractant‐stimulated actin polymerization and migration of mouse neutrophils,^30^, ^51^ as well as for neutrophil recruitment during sterile peritonitis.^51^

## 5 RAC‐GEFS IN NEUTROPHIL ADHESION, MIGRATION AND RECRUITMENT

**Figure 6:**
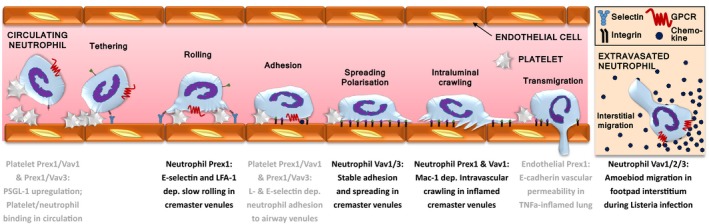
Rac‐GEFs in neutrophil recruitment. Prex1 and Vav Rac‐GEFs control distinct steps of neutrophil recruitment in mice. Neutrophil‐intrinsic roles comprise Prex1‐dependent rolling, Vav1/3‐mediated firm adhesion and spreading, Prex1‐ and Vav1‐dependent intravascular crawling, and Vav1/2/3 interstitial migration. Platelet Prex1/Vav1 controls the binding of platelets to neutrophils in the inflamed circulation and the platelet‐dependent neutrophil adhesion to the airway microvasculature. Endothelial P‐Rex1 controls E‐cadherin‐dependent vascular permeability and neutrophil extravasation during acute lung inflammation

As neutrophil Rac‐GEFs couple Rac‐GTPases to different signalling pathways, it is no surprise that they also control different aspects of neutrophil adhesion, migration and tissue recruitment (Figures 5 and 6). We should note that we focus here on neutrophil‐intrinsic roles of Rac‐GEFs. Neutrophil‐extrinsic roles in recruitment, for example through effects on vascular endothelial cells or platelets, are reviewed in more detail elsewhere.^8^, ^13^

### 5.1 P‐Rex

Isolated Prex1^−/−^ mouse neutrophils show reduced actin polymerization, adhesion and speed of migration upon stimulation of GPCRs, although their directional sensing is preserved.^52^, ^53^, ^54^ Under flow conditions (employed to mimic the shear‐stress encountered by neutrophils within the vasculature), Prex1^−/−^ neutrophils show impaired slow rolling, due to effects on E‐selectin‐mediated activation of the β2‐integrin LFA‐1, as well as reduced Mac‐1 integrin‐dependent crawling on endothelial cells.^55^ In vivo, Prex1^−/−^ mice show similar defects in the E‐selectin and LFA‐1‐dependent slow rolling and Mac‐1‐dependent crawling of neutrophils in the inflamed cremaster vasculature^55^ (Figure 6). Moreover, their neutrophil recruitment is also impaired in sterile peritonitis and during ischaemia reperfusion of the kidney.^52^, ^53^, ^55^

### 5.2 Vav

Vav1^−/−^ mouse neutrophils show impaired fMLP‐stimulated actin polymerization and chemotaxis.^56^ They also show reduced Mac‐1‐dependent crawling under flow conditions, both in vitro and in MIP‐2 inflamed cremaster muscle venules.^57^ Neutrophils from Vav1^−/−^Vav3^−/−^ mice can adhere and chemotax towards fMLP, but show defects in either FcR‐ or integrin‐dependent adhesion and spreading. This is not seen in cells lacking either Vav isoform alone.^58^, ^59^ Furthermore, the firm adhesion of neutrophils to fMLP‐inflamed cremaster muscle venules is reduced in Vav1^−/−^Vav3^−/−^ mice.^59^ Vav1^−/−^Vav2^−/−^ Vav3^−/−^ (Vav‐null) neutrophils show a substantial spreading defect.^54^ Yet surprisingly, neutrophil recruitment is largely normal during sterile peritonitis in Vav1^−/−^ and Vav1^−/−^ Vav3^−/−^ mice,^56^, ^57^, ^59^ upon immune‐complex deposition in the skin or lung in Vav1^−/−^Vav3^−/−^ mice,^60^ and during *S. aureus* infection of the lung in Vav‐null mice.^61^ Despite this relatively normal neutrophil recruitment, the ability of Vav‐null mice to clear pulmonary infections of *P. aeruginosa* or *S. aureus* is strongly impaired.^61^ In addition, these mice show impaired interstitial neutrophil migration during *L. monocytogenes* infection in the footpad, which suggests a prominent role of the Vav family in integrin‐independent amoeboid neutrophil migration.^62^

### 5.3 P‐Rex/Vav cooperation

Although Vav GEFs alone are largely dispensable for neutrophil recruitment, they do cooperate with P‐Rex1 to regulate neutrophil adhesion, migration and tissue recruitment.^13^, ^54^, ^63^ Prex1^−/−^ Vav1^−/−^ neutrophils show reduced cell surface levels of LFA‐1 and Mac‐1 and have more profound defects in fMLP‐stimulated Rac1 and Rac2 activity, adhesion and migration than neutrophils which lack either the P‐Rex or Vav families.^54^, ^63^ Similarly, neutrophil recruitment during sterile peritonitis is more severely impaired in Prex1^−/−^Vav1^−/−^ or Prex1^−/−^Vav3^−/−^mice than in strains that lack either GEF family.^63^ During LPS‐induced pulmonary inflammation, neutrophil transmigration and airway infiltration are impaired in Prex1^−/−^Vav1^−/−^ and Prex1^−/−^Vav3^−/−^ mice, as a result of reduced L‐ and E‐selectin‐mediated adhesion to airway postcapillary venules and ICAM‐1‐dependent slow rolling.^63^ Importantly, however, these in vivo defects are caused largely by neutrophil‐extrinsic roles of the GEFs in platelets which confer platelet‐dependent neutrophil adhesion to the vasculature.^63^

### 5.4 Tiam

The Tiam‐family Rac‐GEF Tiam2 was identified in neutrophils as a target of the transcription factor ATF3. Lentiviral knockdown of Tiam2 in primary mouse neutrophils inhibits chemotaxis, but it increases fMLP‐stimulated actin polymerization and integrin clustering, which suggests that expression of this GEF may limit adhesion rather than promote it as other neutrophils Rac‐GEFs do.^32^ However, the consequences of Tiam2 expression on other neutrophil responses and on recruitment in vivo remain to be investigated. Furthermore, the Tiam2 homologue Tiam1, which is widely expressed, including some types of leucocytes, also remains to be researched in neutrophils.

### 5.5 Pix

One further Dbl‐type GEF is expressed in neutrophils, PIXα, which is known to activate both Rac and the related GTPase Cdc42 in other cell types. During neutrophil chemotaxis, PIXα regulates directional sensing. However, this GEF was shown to activate Cdc42 rather than Rac during chemoattractant signalling.^64^ Interestingly, another study suggested that a complex containing PIXα, the kinase Pak1 and the Arf‐GAP Git2 can regulate the membrane localization and activity of Rac1 under similar conditions.^65^ In general, the substrate specificity of PIXα (Rac vs Cdc42) depends on its dimerization state, with the binding of Gβγ subunits turning the GEF monomeric and Cdc42‐specific.^66^ This considered, it seems likely that PIXα might activate Rac within Gβγ‐independent neutrophil signalling pathways, but this remains to be seen.

### 5.6 DOCK

Murine deficiency in the DOCK‐type Rac‐GEF DOCK2 causes profound defects in neutrophil chemoattractant signalling, thus impairing actin polarization, leading edge formation and migration speed, although these cells retain β2‐integrin‐mediated adhesion and directional sensing.^29^, ^67^ Although deficiency in the DOCK2‐homologue DOCK5 has little effect on its own, combined deficiency exacerbates the migration defect caused by the absence of DOCK2.^68^ The importance of these DOCK GEFs for neutrophil recruitment in vivo remains to be tested. Interestingly, treatment of isolated neutrophils with the small‐molecule DOCK inhibitor CPYPP has similar effects on migration as the mouse knockout.^68^ The efficacy of this inhibitor is currently rather limited (a general problem with Rac‐GEF inhibitors), but it certainly merits further development.

### 5.7 P‐Rex and DOCK in sequence

Finally, there is a possibility that some neutrophil Rac‐GEFs may signal in sequence. Upon GPCR stimulation, P‐Rex1 can activate RhoG as well as Rac1 and Rac2. As active RhoG is an upstream regulator of DOCK2 (through Elmo), P‐Rex1 might signal through RhoG to activate DOCK2.^30^ However, as described above, the actin polymerization and migration defects of Prex1^−/−^ and Dock2^−/−^neutrophils are more varied and severe than those of RhoG^−/−^ neutrophils, which implies that these GEFs signal largely independently of RhoG and each other to generate neutrophil responses.

## 6 CONCLUDING REMARKS

The complexity of Rac‐dependent responses necessitates the tight control mechanisms described here to ensure that neutrophils provide robust antibacterial and antifungal immunity without causing inflammatory disease. Rac‐GEFs are key elements of these tight control mechanisms, each regulating specific aspects of neutrophil adhesion, migration and tissue recruitment. One avenue of research that we expect to be developed over the coming years is the use of live imaging to define how different neutrophil Rac‐GEFs bring about specific subsets of Rac‐dependent cell responses. One intriguing possibility is that different types of GEF activate spatiotemporally distinct subcellular pools of Rac. A valuable tool for this research will be the Rac‐FRET mouse that reports Rac activity^15^ and can be crossed to Rac‐GEF deficient mouse strains. In addition, it is likely that more neutrophil Rac‐GEFs remain to be identified, and others require further characterization. For example, no neutrophil Rac‐GEFs have been identified to date that control the initial tethering step, transendothelial migration or the crossing of the basal lamina and pericyte layer during recruitment. Furthermore, while changes in neutrophil Rac levels or activity clearly cause human immunodeficiencies, it remains to be seen whether altered Rac‐GEFs levels or activity also causes human disease. Such a discovery would be very exciting, as it would open up new avenues of translational research. The idea to fine‐tune Rac activity to a level that preserves immunity while combating neutrophil‐mediated inflammatory conditions is appealing. Yet, direct inhibition of Rac or Rac‐GEFs is not trivial, as these proteins work through protein/protein interactions, and most current inhibitors are quite inefficient and lack specificity. The targeting of cell surface adhesion molecules and receptors to which Rac‐GEFs are coupled seems to be a more promising approach, which has already started to yield results in animal models of inflammatory disease. The identification of new roles for Rac‐GEFs in controlling the surface levels or activity of neutrophil adhesion molecules and receptors will be another important area of future research that will hopefully pave the way for novel rationally designed anti‐inflammatory therapies.
